# A Narrative Review on ECMO as a Bridge to Transplantation: Optimising Outcomes in Heart and Lung Failure

**DOI:** 10.7759/cureus.100650

**Published:** 2026-01-02

**Authors:** Pragnya Bandari, Yezen M.H. Alnasiri, Amna Ahsan, Hamritha Kokila Kripa Manoharan, Syam P Maharaj, Venkata Yashashwini Maram Reddy, Aliya Shaju Shahul Hameed, Swetha Chinthala, Chanza Shaikh, Kausar Bano, Ramsha Ali

**Affiliations:** 1 Medicine, Malla Reddy Medical College for Women, Hyderabad, IND; 2 Medicine, Humanitas University, Milan, ITA; 3 Cardiology, Queen Elizabeth Hospital, Birmingham, GBR; 4 Medicine, Pondicherry Institute of Medical Sciences and Research, Pondicherry, IND; 5 Critical Care, San Fernando General Hospital, San Fernando, TTO; 6 Medicine, Guntur Medical College, Guntur, IND; 7 Medicine, East European University, Tbilisi, GEO; 8 Medicine, Davao Medical School Foundation, Davao, PHL; 9 Medicine, LSU Health Shreveport, Shreveport, USA; 10 Medicine, Katihar Medical College, Katihar, IND; 11 Medicine and Surgery, Peoples University of Medical and Health Sciences, Hyderabad, PAK

**Keywords:** bridge to transplantation, ecmo, extracorporeal membrane oxygenation support, heart failure, lung failure

## Abstract

Extracorporeal membrane oxygenation (ECMO) provides short-term cardiopulmonary support for patients with end-stage heart and lung failure and is increasingly used as a bridge to transplantation. Its expanding clinical role has prompted the need to clarify how patient selection, timing of initiation, and complication risks influence transplant eligibility and outcomes. This review aims to evaluate contemporary evidence on ECMO as a bridge to heart and lung transplantation by outlining selection criteria, analysing the timing of intervention, summarising survival and complication data, and identifying the limitations that prevent consistent clinical standardisation.

For the selection of articles, a narrative review approach was used. Articles were screened from PubMed and Rayyan, restricted to English-language studies published within the past 15 years, using the search terms “ECMO”, “heart failure”, and “lung failure”. Paediatric studies and non-English literature were excluded. Across the literature, patient selection is guided by absolute contraindications (e.g., chronic multiorgan dysfunction, unrecoverable primary cardiac disease) and relative contraindications such as advanced age. Early initiation of ECMO is consistently associated with improved survival. The venoarterial (VA)-ECMO and venovenous (VV)-ECMO provide critical cardiac and respiratory support with varying survival outcomes; however, the therapy is associated with substantial clinical risks, including renal failure, significant bleeding, systemic infection, and vascular complications such as stroke or limb ischaemia. Prolonged bridging due to donor shortages further complicates outcomes and limits comparability across centres. Thus, it reinforces that ECMO is a crucial bridge to heart and lung transplantation. However, its effectiveness depends on precise patient selection, early and standardised initiation strategies, and unified clinical guidelines. Improved standardisation and consistent reporting are essential to optimise outcomes and strengthen long-term post-transplant evidence.

## Introduction and background

Extracorporeal membrane oxygenation (ECMO) is a temporary form of life support used in critically ill patients with severe cardiac and/or respiratory failure who do not respond to conventional therapies. It provides extracorporeal gas exchange and circulatory support, stabilising patients while definitive treatment, most commonly heart or lung transplantation, is pursued. The ECMO is broadly categorised into two configurations based on clinical indication. Venoarterial (VA)-ECMO provides both circulatory and respiratory support and is primarily used in patients with cardiac failure or cardiogenic shock [1]. In contrast, venovenous (VV)-ECMO provides respiratory support alone in patients with preserved cardiac function [1]. The VV-ECMO has been successfully used as a bridge to lung transplantation in patients with acute respiratory distress syndrome (ARDS) [2]. In cardiac failure, VA-ECMO provides circulatory support to maintain stability while awaiting heart transplantation [1,3,4]. This narrative review focuses on the rationale for ECMO bridging, current clinical barriers, its relevance in practice, and gaps in the existing evidence.

Rationale for ECMO bridging 

The primary rationale for ECMO is to provide temporary life support in patients with severe cardiopulmonary failure, creating a bridge while definitive treatment, most commonly heart or lung transplantation, is arranged. The ECMO also supports haemodynamic stability in patients with refractory cardiogenic shock, including cases secondary to acute myocardial infarction [3,4]. The use of ECMO has expanded beyond emergency support. Expert consensus supports ECMO in preoperative, intraoperative, and postoperative settings [5,6]. It is applied across diverse clinical scenarios. In respiratory failure, VV-ECMO has served as a bridge to lung transplantation, including in complex cases such as ARDS [2,7,8]. It can also be combined with other therapies, such as continuous renal replacement therapy, in patients with multi-organ failure [9].

Current barriers

Access to definitive treatments such as heart or lung transplantation for patients stabilised on ECMO is often limited by organ scarcity and the absence of standardised allocation systems [10,11]. Global variability in allocation policies leads to disparities in access. For example, the United States prioritises patients based on clinical urgency and post-transplant survival probability, whereas South Korea’s allocation is influenced by geographic regions [11]. These limitations may result in prolonged ECMO support, which can increase the risk of complications such as infection, bleeding, stroke, pulmonary oedema, and vascular access-related interventions [10]. Prolonged support can further affect clinical stability and delay transplantation.

Clinical relevance and survival outcomes

Extracorporeal membrane oxygenation is a life-saving therapy that can be applied in various clinical settings. However, its broader use is limited by the lack of standardised protocols and robust long-term outcome data [11]. Survival outcomes in hospitalised patients are generally favourable [12], but vary according to several factors. Patient-related factors include age and pre-existing health conditions, with younger patients demonstrating better outcomes in conditions such as fulminant myocarditis [7,13]. Intervention-related factors, such as patient selection criteria and timing of ECMO initiation, also influence outcomes [12]. Additionally, higher-volume treatment centres are associated with improved survival rates [13].

Despite evidence from case reports and observational studies supporting ECMO as a bridge to transplantation, high-quality data on long-term survival, optimal cannulation techniques, and best practices for patient selection remain limited. Standardised guidelines are needed to optimise ECMO use and improve clinical outcomes [10,14,15].

## Review

Methodology

Database Search and Strategy

The principal literature search was conducted through the utilization of the PubMed and Rayyan database repositories. The search was initially performed on May 2, 2025, and was re-searched and updated on September 18, 2025, to capture the most recent evidence. The search strategy employed a combination of key terms and Boolean operators. The full PubMed search string was “ECMO,” “bridge to transplantation,” “heart failure,” “lung failure,” and “extracorporeal support.” The equivalent strategy was adapted for the Rayyan database repository.

Eligibility and Inclusion-Exclusion Criteria

Only English-published literature concerning the adult patient population and published within the last 15 years (2009 and onwards) was taken into consideration. Articles selected were heterogeneous, comprising narrative reviews, systematic reviews, meta-analyses, expert consensus documents, clinical guidelines, and interesting case reports, all of which were pertinent to clinical evidence and professional opinion regarding the use of ECMO as a bridge strategy.

Studies were excluded if they met any of the following criteria: non-English publications, publications older than 15 years, publications predominantly concerning the utilization of ECMO with organs other than the heart or lungs (except when considering an applicable cardiopulmonary comorbid disease state), or a clinical situation wherein ECMO was being utilized purely as an adjunct to postoperative recovery with no reference to transplantation.

Study Selection and Screening

The initial search across both databases yielded a total of 245 records. All records were imported into the Rayyan platform for screening. After automatic and manual removal of duplicates, 62 records were excluded, leaving 183 records for abstract and title screening. Two independent reviewers screened the titles and abstracts against the eligibility criteria. At this stage, 63 records were excluded for not meeting the population, language, or date criteria. The full text of the remaining 120 articles was retrieved and assessed for eligibility. Of these 120, 49 articles were excluded during full-text review primarily due to a lack of transplantation context or a focus on non-cardiopulmonary use, leaving a total of 71 articles to be included in this review. Disagreements regarding inclusion were resolved by consensus.

Quality Assessment and Synthesis

While a formal risk-of-bias appraisal was not performed, as this is a narrative review, a hierarchy of evidence was employed during synthesis. Priority was given to information derived from high-level evidence, such as systematic reviews, meta-analyses, and expert consensus documents, when substantiating conclusions. This approach was taken to mitigate potential bias and enhance the reliability of the clinical narrative.

Finally, the remaining 71 original, cited references were utilized to substantiate the conclusions arrived at in this narrative review. This study was conducted as a narrative review, and no quantitative synthesis or statistical analyses (including meta-analysis or meta-regression) were planned or performed. Additionally, it is designed as a narrative review, synthesizing heterogeneous clinical and expert evidence. Accordingly, no formal risk-of-bias assessment tool or quantitative synthesis was applied.

Selection criteria and patient candidacy

The suitability of ECMO as a bridge for heart or lung transplant requires careful, multidisciplinary consideration of the patient's underlying disease, physiological status, and potential for rehabilitation, while excluding prohibitive contraindications [16]. Extracorporeal membrane oxygenation is generally considered when conventional medical therapies, including mechanical ventilation, have failed to manage severe respiratory or cardiac failure [15]. The underlying condition should be refractory primary heart or lung failure amenable to transplantation, though multiple organ failure can occur and impact outcomes [17, 18].

**Figure 1:**
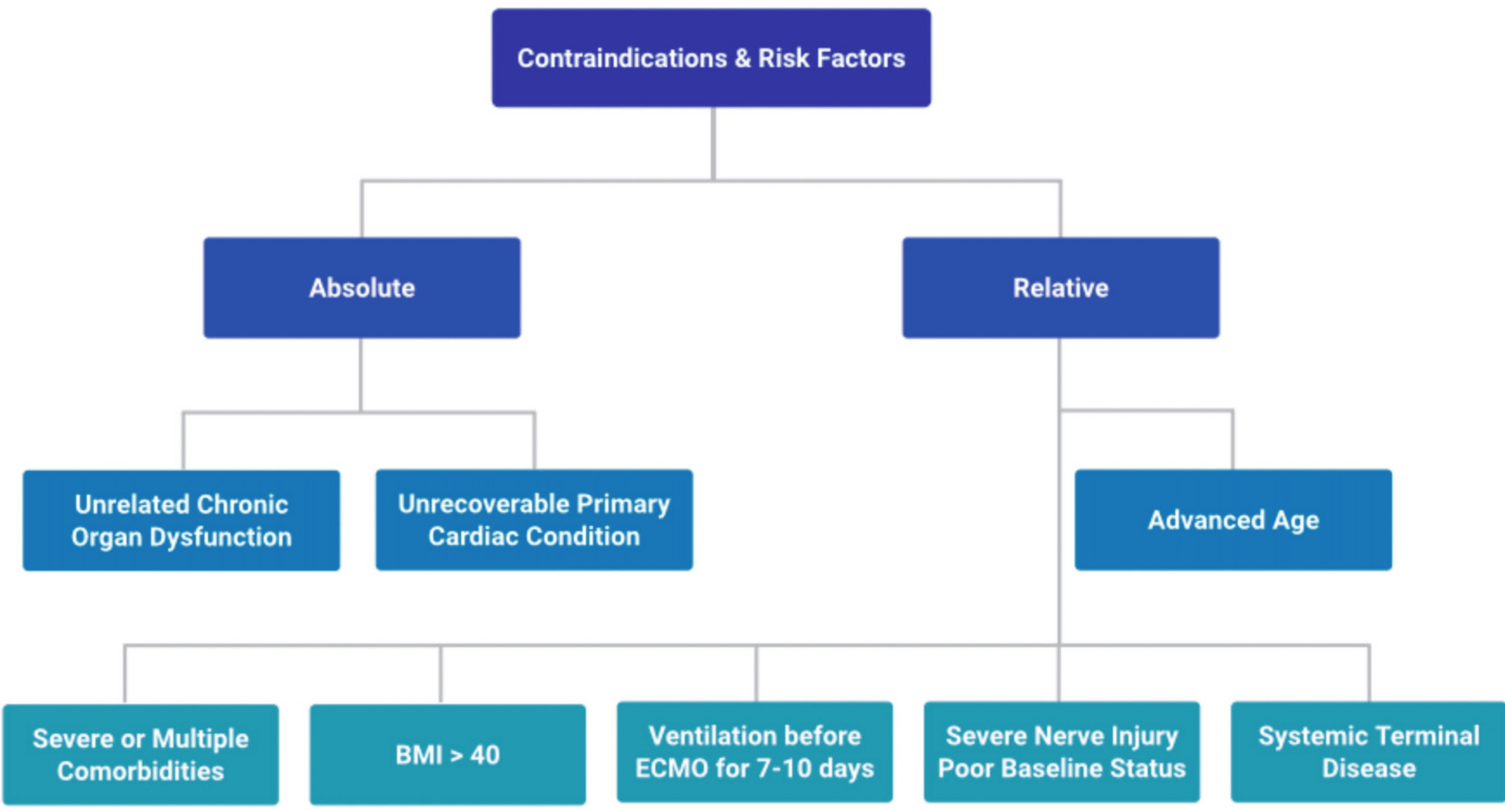
Contraindications and risk factors for ECMO-bridged transplant recipients Flowchart of absolute and relative contraindications for selection of patients for ECMO therapy created by author AlNasiri using BioRender (Toronto, ON, CAN). ECMO: Extracorporeal membrane oxygenation

A critical aspect of ECMO bridging is that eligibility is not static. Some patients initially deemed unsuitable may stabilize on ECMO and become transplant candidates or even recover sufficiently to be weaned from support [18, 19]. Continuous and rigorous assessment of organ function, potential for rehabilitation, and overall clinical trajectory while on ECMO is crucial for determining ongoing eligibility and optimizing the timing of transplantation [18, 19]. Furthermore, absolute contraindications include active, refractory malignancies, severe neurological injuries, and, if patients are not transplant candidates, irreversible primary cardiac conditions, as the presence of such signs would likely render ECMO futile (Figure 1) [16,20,21].

Extracorporeal membrane oxygenation modality selection depends on the primary organ failure. Respiratory support with VV-ECMO is indicated for isolated critical respiratory failure, such as refractory ARDS (including secondary to COVID-19), particularly for patients with isolated hypoxia and/or hypercapnia without significant hemodynamic instability or RV dysfunction. Transplantation is generally reserved for patients with no pulmonary improvement who remain fully dependent on support [5,15,17,18,20,22]. Cardiorespiratory support with VA-ECMO is the preferred strategy for patients with severe cardiac failure refractory to maximal medical therapy, including those with acute cardiogenic shock (CS) from acute myocardial infarction, severe biventricular failure, acute right ventricle (RV) failure, fulminant myocarditis, and post-cardiotomy shock [1,23,24,25]. Venoarterial ECMO is also used when lung failure presents with hemodynamic instability. Venopulmonary configurations (e.g., ProtekDuo cannula) can also benefit patients with lung failure and concomitant RV failure [15].

Absolute Contraindications and Risk Factors of Selection

The Extracorporeal Life Support Organization (ELSO) guidelines and clinical experience highlight several factors associated with increased risk or futility (Table 1).

**Table 1:** Absolute contraindications and risk factors ARDS: Acute respiratory distress syndrome, CS: Cardiogenic shock, PaO2: Partial pressure of oxygen, FiO2: Fraction of inspired oxygen, PaCO2: Partial pressure of carbon dioxide

Category	Factors	Evidence quality and comment
Absolute contraindications	Significant chronic organ dysfunction unrelated to the acute presentation (e.g., advanced cirrhosis, severe emphysema, pre-existing end-stage renal failure); unrecoverable primary cardiac condition if the patient is not a candidate for transplantation or a durable mechanical circulatory support device [16].	These factors are generally considered to render ECMO futile, leading to a "bridge to nowhere" [1,4,26].
Major risk factors	Advanced age [9,19,27,28]; presence of multiple or severe comorbidities including multi organ dysfunction [29]; BMI >40 kg/m2 (especially for heart transplant) [23,30]; ventilation >7-10 days before ECMO [15,17,19]; active, non-treatable malignancies or other terminal systemic diseases [30]; severe and irreversible neurological injury or poor baseline functional status [15,17,19]	Current evidence suggests these recipient characteristics are associated with increased post-transplant mortality, with advanced age being consistently identified as a major risk factor [9,19,27,28]
ARDS-specific risk factors	Severe hypoxemia (partial pressure of oxygen (PaO2)​/fraction of inspired oxygen (FiO2)​ <50-80 mmHg for several hours) or severe hypercapnic acidosis (pH<7.25 with partial pressure of carbon dioxide (PaCO2) ​≥60 mmHg) despite optimized conventional management [30]	
Futility	Multi-organ failure or catastrophic neurological injury that is not reversible [31]; inability to pass weaning trials [31]; baseline condition (ARDS, CS) does not improve after prolonged therapy, rendering the patient unsuitable for transplant [32]	Determining futility remains a profound ethical challenge when the medical team recognizes the likely outcome, but the family maintains hope [33]

Risks and complications

**Figure 2:**
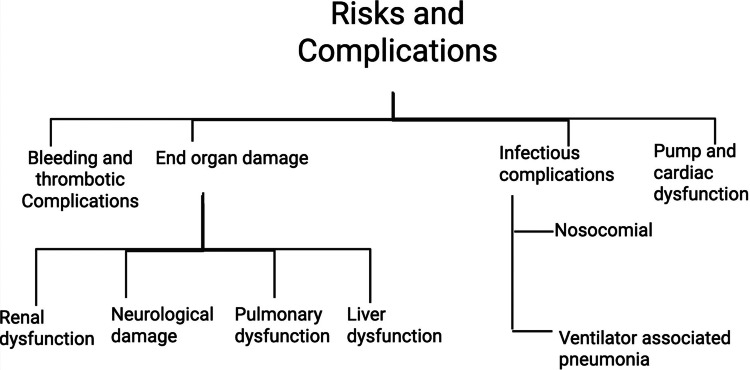
Risks and complications of patients on ECMO therapy Flowchart entailing bleeding, thrombotic, end-organ damage, infectious complications, and pump-related complications created by author Bandari using BioRender. ECMO: Extracorporeal membrane oxygenation

The use of ECMO is likened to a double-edged sword, significantly increasing survival for severely ill patients but carrying a high incidence of risks that contribute to morbidity and mortality (Figure 2) [4,34-36].

Hemorrhagic and thrombotic complications are major, often controversial, complications related to anticoagulation management [34,35,37]. Bleeding is a chief cause of mortality [37] and is reported in up to 38% of patients, with intracranial bleeding being a greatly feared complication [34,37]. Thrombosis is also frequent, with rates reported between 20% (for low molecular weight heparin (LMWH)) and 50% (for unfractionated heparin (UFH)) [34,35]. Controversy in anticoagulation exists; evidence suggests a complex trade-off, where UFH-treated patients had lower bleeding events (12.5% vs. 22.7%) [38], yet UFH is associated with higher thrombotic risk (50% vs. 20% for LMWH) [34]. Current evidence suggests that reducing UFH dosing in lung transplantation may decrease blood loss while preserving thrombotic safety [6].

Organ dysfunction and end-organ damage associated with critical illness are often worsened by prolonged ECMO runs (>250 hours) [35,36]. Renal dysfunction occurs in 20% to 50% of patients and is more prevalent in VA-ECMO than VV-ECMO, often requiring continuous renal replacement therapy (CRRT) [35,36]. Neurological injury, such as stroke in 4% of ECMO recipients, is mostly encountered in VA-ECMO due to arterial embolic events [34,35]. Liver dysfunction can result from RV failure-induced hepatic congestion, leading to elevated liver enzymes and metabolic instability [35].

Pump-related and specific complications related to VA-ECMO are closely related to cardiac support and arterial access. Elevated left ventricular (LV) afterload is a major concern that can lead to LV distension, pulmonary edema, and further myocardial injury, often necessitating venting maneuvers (e.g., Impella insertion) to avoid these life-threatening consequences [14,32,39]. Pump-specific complications include pump thrombosis due to heparin-induced thrombocytopenia (HIT) [40] or hemolysis from femoral Impella insertion [39]. Limb ischemia is reported in 5% of patients [34,35]. Decannulation issues can result in significant arterial bleeding (4.1%) requiring emergency vascular repair (3.7%) [41]. Infectious complications are a primary risk, seen in 21% of patients, especially with extended ECMO exposure [4,1,35]. The VV-ECMO patients face a greater risk of ventilator-associated pneumonia (VAP) from prolonged mechanical ventilation [35,41] while VA-ECMO patients show higher bloodstream infection rates [35,41].

Results

Survival Outcomes

**Figure 3:**
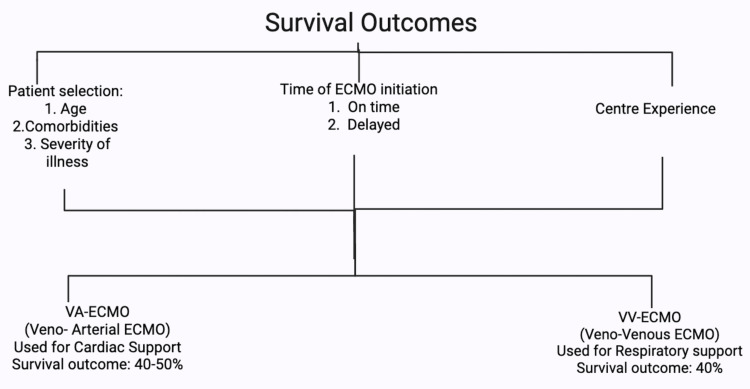
Factors affecting survival outcomes of patients on ECMO Flowchart entailing factors of patient criteria, time of initiation, and centre experience factors, including the difference of outcomes between VA-ECMO and VV-ECMO, created by author Bandari using BioRender. ECMO: Extracorporeal membrane oxygenation

Current evidence suggests ECMO, while providing a necessary bridge for severely unstable patients to undergo transplantation, is associated with a high short-term mortality (33%) and a one-year mortality of 50% [36]. However, almost 50% of critically ill adult patients receiving ECMO survive up to hospital discharge (Figure 3) [42]. Table 2 lists the factors influencing survival outcomes. 

**Table 2:** Factors influencing survival outcomes ECMO: Extracorporeal membrane oxygenation, VV-ECMO: Venovenous ECMO, VA: Venoarterial ECMO

Factor	Finding	Controversy/Critical comment
Patient selection	Survival is significantly influenced by age, underlying conditions, and severity of illness [43]	Rigorous selection is key, as advanced age, multiple comorbidities, and prolonged pre-ECMO ventilation consistently correlate with poor prognosis and elevated mortality [9,19,23].
Timing of initiation	Early initiation in appropriate candidates may improve survival, whereas delayed initiation often correlates with poorer outcomes due to potential irreversible end-organ damage [3,44,45]	The optimal timing of ECMO initiation is a highly discussed area. Early cannulation can decrease short-term mortality [1], but it must be balanced against the risk of unnecessary support. Delaying the intervention is associated with lower survival outcomes due to potential end-organ damage, which would make the patient an unsuitable candidate for transplantation [9,3,23].
ECMO modality	Survival to discharge averages ~60% for VV-ECMO (respiratory failure) but varies widely for VA-ECMO, often 40% to 50 [43]	Current evidence suggests transplantation provides a survival benefit for listed patients on VA-ECMO, though post-transplant survival remains inferior to that of non-ECMO patients [46].
Center experience	High-volume ECMO centers with experienced multidisciplinary teams tend to report superior survival rates [47-49]	This "center effect" suggests that outcomes are not solely patient-dependent but also linked to optimized protocols and comprehensive care [23,50,51]. Given the complexity and resource intensity, high-volume and experienced centers will be better equipped to tackle the challenges that come with such therapies, as well as having well-trained response teams and multidisciplinary approaches [50,51].
Post-transplant outcomes	Patients who undergo ECMO for late graft failure have worse long-term outcomes than those with early graft failure; rescue graft support for late failure was universally unsuccessful [52]	Controversy exists regarding long-term post-transplant outcomes, especially given the universally poor outcomes for late graft failure rescue [52].

There was no difference in mortality on pump support compared with post-transplant mortality among those bridged from ECMO to left ventricular assist device (LVAD) or hypertension [53]. In adults with CS receiving VA-ECMO without concomitant LV mechanical unloading, the use of partial (<2.0 L/min/m²) versus full ECMO flow index was associated with lower 30-day in-hospital mortality [54].

Respiratory ECMO Survival Prediction Score

The respiratory ECMO survival prediction (RESP) score [49] was developed by ELSO and the Department of Intensive Care at The Alfred Hospital, Melbourne, Australia. It is designed to predict survival for adult patients undergoing ECMO for respiratory failure. It should not be considered for patients who are not on ECMO or as a substitute for clinical assessment.

Allocation systems for ECMO in heart and lung transplant

**Figure 4:**
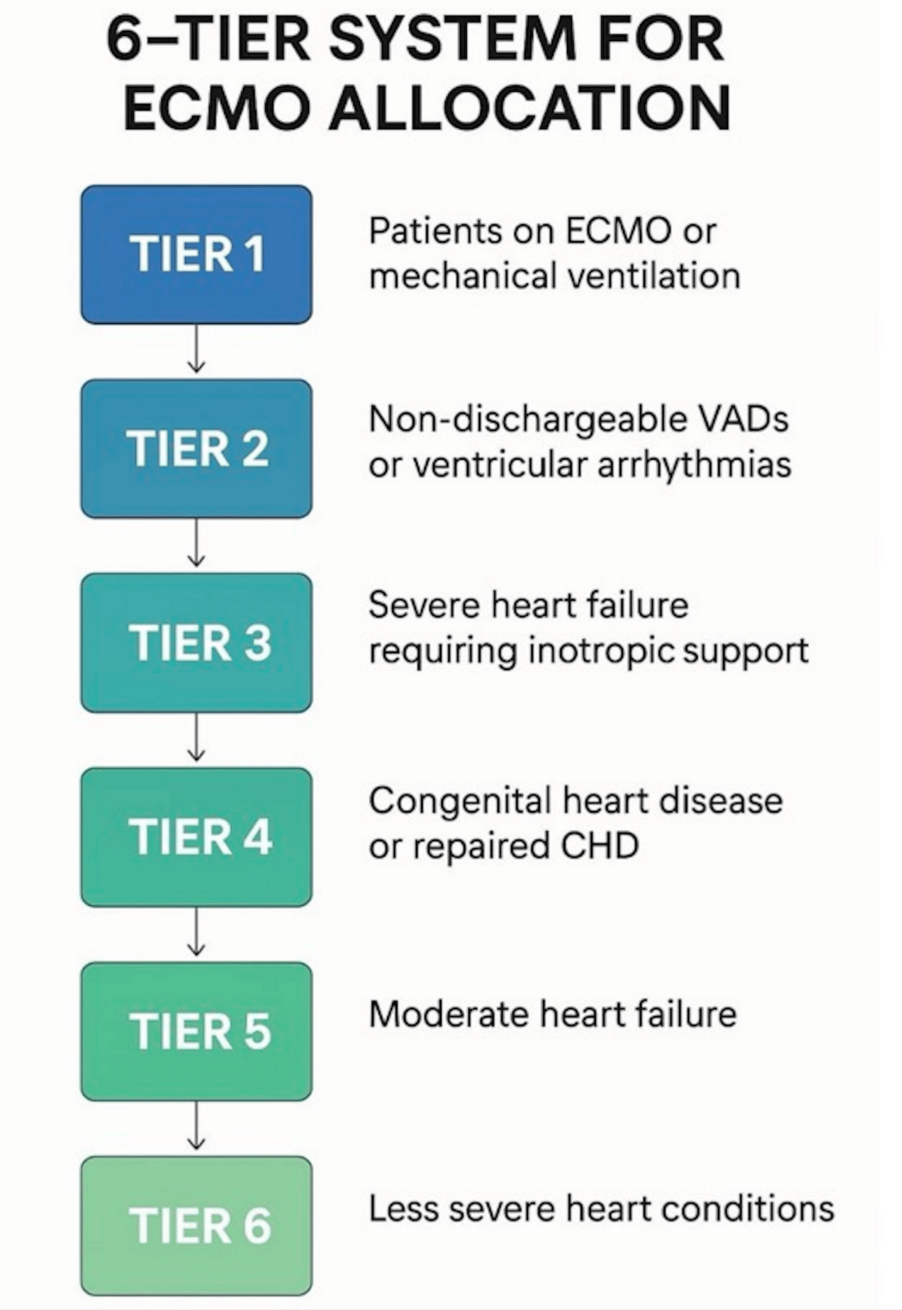
Stratification of patients for allocation systems of ECMO Flowchart of the UNOS-based six-tier allocation system based on the condition and criticality of the patient, created by author Bandari using BioRender. ECMO: Extracorporeal membrane oxygenation, UNOS: United Network for Organ Sharing, VAD: Ventricular assist device, CHD: Congestive heart disease

Allocation criteria determine which patients on ECMO are prioritized for limited donor organs. This is done by balancing clinical urgency, risk of death without transplant, utility, and the likelihood of post-transplant survival, governed by national policies such as the United Network for Organ Sharing (UNOS)/Organ Procurement and Transplantation Network (OPTN) and international consensus like the International Society for Heart and Lung Transplantation (ISHLT) (Figure 4).

Heart transplantation under the OPTN/UNOS adult heart allocation system designates candidates on VA-ECMO as status 1, the highest priority for transplant, provided they are hospitalized at a transplant center and meet criteria for CS [55,56]. The ISHLT 2024 guidelines emphasize that patients on temporary mechanical circulatory support, including ECMO, should be comprehensively evaluated for comorbidities, frailty, infection status, and rehabilitation status before being listed [57]. The controversy in allocation granularity is that while VA-ECMO is prioritized, ISHLT highlights that LVAD patients with complications may have waitlist mortality comparable to or greater than ECMO patients, thus arguing the system must allow sufficient granularity to reflect this complex risk [58].

Lung transplantation under the lung allocation score (LAS) determines priority by integrating waitlist urgency and projected post-transplantation survival. Extracorporeal membrane oxygenation support increases LAS due to its strong association with waitlist mortality [59]. However, recent analysis notes that the LAS can underestimate risk for certain ECMO patients, especially those with prolonged support or additional organ dysfunction, impacting projected post-transplant and waitlist survival [60]. The ISHLT consensus statements recommend that ECMO as a bridge to lung transplantation be limited to patients with acceptable transplant candidacy [61]. Ambulatory ECMO patients achieve superior clinical outcomes when compared to those intubated, sedated, or bed-bound [62].

The ELSO registry data for lung transplantation consistently show an expansion of ECMO use as a bridge to transplant (BTT), with recent outcomes indicating improved survival rates, particularly when protocols emphasize careful patient selection and early mobility (ambulatory ECMO) to minimize deconditioning [54,62]. The key principles across both allocation systems are listed in Table 3.

**Table 3:** Key principles across both heart and lung transplantation systems ECMO: Extracorporeal membrane oxygenation

Parameter	Condition
Clinical urgency is recognised	ECMO patients are prioritized due to their high risk of death without transplant [55, 59]
Utility is protected	Only candidates with reasonable post-transplant survival likelihood are listed [57,61]
Transparency and fairness	Explicit, standardised criterion applied nationally [55, 63]
Reassessment is mandatory	Patients on prolonged ECMO require regular multidisciplinary evaluation to determine ongoing candidacy [57,58]

Discussion

The role of ECMO has broadened beyond basic life support to address multiple clinical crises. It provides hemodynamic stability in refractory cardiogenic shock (VA-ECMO) and aids in cases of severe ARDS (VV-ECMO), including those caused by COVID-19 [2, 4]. Furthermore, it allows for multi-organ support by combining with therapies like continuous venovenous hemodiafiltration for complex heart and renal failure cases [9].

The specific modality of ECMO dictates the focused clinical management required. For respiratory failure, the success of the VV-ECMO modality relies heavily on a 'bridge-to-rehabilitation' strategy, highlighting the strong correlation between preoperative functional status (e.g., ambulatory ECMO) and improved post-transplantation prognosis [4, 20]. In contrast, VA-ECMO for CS carries the unique risk of elevated LV afterload and subsequent LV distension, which compromises recovery and increases pulmonary edema. Venoarterial-ECMO increases LV afterload due to the retrograde high-velocity flow ejected into the arterial system, opposing native cardiac output. This impedance elevates LV end-systolic pressure and impairs ejection fraction, leading to LV distension, acute pulmonary venous congestion, and potentially exacerbating myocardial stunning or injury [64].T his hemodynamic complexity necessitates immediate LV unloading maneuvers to manage the complication and maximize the chances of success [14,39]. Across both modalities, prolonged duration of support remains the primary risk factor for secondary complications, particularly VAP and bloodstream infections [20,35].

The current organ allocation frameworks, such as the UNOS and LAS systems, correctly categorize ECMO patients as having the utmost clinical urgency [65]. However, this review demonstrates a critical weakness in the nuanced risk stratification embedded within these systems [66]. By assigning the highest priority status to ECMO patients, the system often gives equal priority to individuals with vastly different clinical trajectories, from relatively stable LVAD patients with complications to rapidly deteriorating VA-ECMO patients. This leads to a tension where the allocation priority (sickest patient) may not align with transplant utility (best chance of long-term survival) [66]. This resource allocation conflict compounds the ethical dilemma of the 'bridge to nowhere' [1,26], demanding careful consideration for the ethical continuance versus the morally difficult decision to withdraw costly, resource-intensive support when futility is established [33,67].

Limitations and Ethical Challenges of ECMO

The use of ECMO as a bridge is constantly tested against situations of failure, leading to systemic, ethical, and clinical conundrums. The greatest failure of ECMO is the 'bridge to nowhere' scenario, where transplant is impossible due to irreversible deterioration, inability to locate a compatible donor organ, or a catastrophic event [1,26]. This constitutes medical futility, where continued therapy only prolongs the process of dying [4]. It is a profound ethical challenge, as the medical team recognizes the futility, while the family may view continuation as hope [33]. Failure is typically signified by irreversible multi-organ failure or catastrophic neurological injury [31]; inability to pass weaning trials; demonstration that the heart or lungs cannot work independently [31]; or failure of the baseline condition (e.g., severe ARDS or CS) to improve after prolonged ECMO, rendering the patient unsuitable for transplantation [32]. Aggressive, urgent ECMO initiation often forecloses comprehensive discussion with the patient/family regarding risks and the potential for a 'bridge to nowhere' [33].

Extracorporeal membrane oxygenation is a highly costly and resource-intensive therapy [67]. The use of sparse resources (hospital staff, funding, donor organs) on a patient with a low probability of transplant or survival raises ethical concerns regarding resource allocation, potentially denying access to another critically ill patient [68]. Patterns of clinical failure for heart candidates (VA-ECMO) include LV distension (due to retrograde flow) [32], which triggers pulmonary edema and increases myocardial stunning. For lung candidates (VV-ECMO), failure is often correlated with the duration of ECMO support, as longer durations increase the incidence of infection (e.g., VAP), potentially rendering the patient a non-viable candidate for transplantation [33].

Future Aspects and Research Priorities

Major challenges remain, as ECMO still carries a high risk of complications, especially bleeding (a chief cause of mortality) and infection [37]. Although survival has improved, prospective long-term research is required to delineate the long-term effects of ECMO on graft function, quality of life, and chronic complications [43]. Research has also highlighted disparities in access to ECMO based on demographics like gender, insurance coverage, and socioeconomics [32,69]. Race and ethnicity also significantly impact ECMO care; studies confirm that minority groups face systemic barriers leading to reduced access and are associated with higher rates of post-treatment complications like acute kidney injury. This underscores that the observed disparities are driven by structural factors, such as insurance and quality of preventative care, rather than biological differences [70]. Future efforts should focus on precision-guided patient selection by creating advanced, personalized predictive tools that incorporate clinical, social, psychological, and demographic elements to ensure equitable access to suitable patients [32]. Innovative prototypes driven by miniaturization (e.g., polymethylpenthene oxygenators) and enhanced monitoring (e.g., integrated fiber-optic sensors), leading to 'smart ECMO' systems. This includes strategies like using venting maneuvers (e.g., Impella insertion) to manage life-threatening LV distension in VA-ECMO [14,39], and promoting 'awake and ambulatory' practice with portable systems to facilitate aggressive physical therapy [32,33,61]. Technological development should focus on efficient, smaller ECMO systems with superior safeguarding capabilities, alongside standard anticoagulation protocols to decrease bleeding and thrombosis [71]. The impact of successful 'awake and ambulatory' practice supports further promise in portable ECMO technology to allow early and aggressive physical therapy [33,32]. Finally, systemic improvement requires advancing the multidisciplinary approach demonstrated by high-volume centers and establishing standardized training and referral protocols to ensure equitable access to first-class facilities [50,51]. The imperative is to transition ECMO from a reactive, high-risk intervention to an equitable, precision-guided therapy, optimizing selection and technology to redefine the landscape of transplant candidacy and long-term survival [33,72].

## Conclusions

Extracorporeal membrane oxygenation represents a crucial yet intricate bridge to heart and lung transplantation, capable of salvaging patients from near-mortality with a considerable hospital discharge potential by delivering necessary cardiopulmonary support. However, this lifesaving therapy is plagued by excessively high costs of complications and the persistent moral dilemma of the 'bridge to nowhere.' Maximizing results is contingent upon a concerted emphasis on vigorous patient selection (favoring early onset and rehabilitation potential), the implementation of the multidisciplinary, high-volume center method, and the creation of sophisticated, personalized predictive models. Ultimately, the future of ECMO must transition from mere life sustainment to precision-guided therapy to ensure equitable access and optimal longer-term survival post-transplant. Our narrative review uniquely provides the first systematic synthesis of the complex clinical, ethical, and logistical failure points of ECMO as a bridge, specifically focusing on selection contraindications, futility determination, and the inherent tensions within the UNOS/LAS organ allocation systems. We offer an essential framework by linking patient-specific risk factors to systemic allocation challenges, which is crucial for refining policy and minimizing tragic 'bridge to nowhere' scenarios.

## References

[1] Abu Akel M, Shaul AA, Goldenberg GR (2022). Combined mechanical circulatory support for ventricular fibrillation in left ventricular assist device patient. ESC Heart Fail.

[2] Foessleitner P, Hoetzenecker K, Benazzo A, Klebermass-Schrehof K, Scharrer A, Kiss H, Farr A (2021). Bilateral lung transplantation during pregnancy after ECMO for influenza-A caused ARDS. Am J Transplant.

[3] Choi KH, Yang JH, Hong D (2020). Optimal timing of venoarterial-extracorporeal membrane oxygenation in acute myocardial infarction patients suffering from refractory cardiogenic shock. Circ J.

[4] Chou HW, Wang CH, Lin LY, Chi NH, Chou NK, Yu HY, Chen YS (2020). Prognostic factors for heart recovery in adult patients with acute fulminant myocarditis and cardiogenic shock supported with extracorporeal membrane oxygenation. J Crit Care.

[5] Hartwig M, van Berkel V, Bharat A (2023). The American Association for Thoracic Surgery (AATS) 2022 expert consensus document: the use of mechanical circulatory support in lung transplantation. J Thorac Cardiovasc Surg.

[6] Di Nardo M, Ahmad AH, Merli P (2022). Extracorporeal membrane oxygenation in children receiving haematopoietic cell transplantation and immune effector cell therapy: an international and multidisciplinary consensus statement. Lancet Child Adolesc Health.

[7] Vadlamudi GD, Keerthy M, Goyert G (2022). Postpartum bilateral lung transplantation in COVID-19 associated respiratory failure. BMJ Case Rep.

[8] Wang B, Huang J, Hsin M, Chen J, Lin H (2021). First lung transplant in Wuhan for a critical and elderly COVID-19 patient. Immun Inflamm Dis.

[9] Silva DT, Dantas C, Santos AS (2021). Combined lung-kidney transplantation: first case in Portugal. Respir Med Case Rep.

[10] Sert D, Kervan U, Kocabeyoglu S (2022). Prolonged extracorporeal membrane oxygenation support as a bridge to heart transplant. Exp Clin Transplant.

[11] Kim KH (2023). Optimizing outcomes for post-ECMO heart transplant patients in South Korea: addressing multi-organ failure and allocation challenges. Int J Heart Fail.

[12] Xiong J, Zhang L, Bao L (2020). Complications and mortality of venovenous extracorporeal membrane oxygenation in the treatment of neonatal respiratory failure: a systematic review and meta-analysis. BMC Pulm Med.

[13] Tran A, Fernando SM, Rochwerg B (2023). Prognostic factors associated with mortality among patients receiving venovenous extracorporeal membrane oxygenation for COVID-19: a systematic review and meta-analysis. Lancet Respir Med.

[14] Randhawa VK, Hoffman K, Bock A (2020). Impella RP as a bridge to cardiac transplant for refractory late right ventricular failure in setting of left ventricular assist device. ESC Heart Fail.

[15] Capoccia M, Brewer JM, Rackauskas M (2024). Outcome of veno-pulmonary extracorporeal life support in lung transplantation using Protekduo cannula: a systematic review and description of configurations. J Clin Med.

[16] Zotzmann V, Wengenmayer T, Lang CN (2021). Case report: refusal of an veno-arterial extracorporeal membrane oxygenation due to malignant disease? -— an extremely rare form of cardiac involvement in acute myeloid leukemia. Front Med (Lausanne).

[17] Gogia P, Attawar S, Singh V, Bhatnagar T, Sharma S, Batra K, Khare S (2021). Lung transplantation for post-COVID-19 pulmonary fibrosis. Respirol Case Rep.

[18] Magnusson JM, Silverborn M, Broomé M, Riise GC, Dellgren G (2022). Long-term extracorporeal membrane oxygenation bridge to lung transplantation after COVID-19. Ann Thorac Surg.

[19] Zhu Y, Zeng F, Lan MJ, Liang JS, Cai LY, Gu PP, Guo LY (2024). Prognostic factors in lung transplantation after extracorporeal membrane oxygenation bridging therapy: a systematic review and meta-analysis. J Thorac Dis.

[20] Ko RE, Oh DK, Choi SM (2022). Lung transplantation for severe COVID-19-related ARDS. Ther Adv Respir Dis.

[21] Howick V JF, Rezkalla JA, Tilbury T (2023). Venoarterial extracorporeal membrane oxygenation after autologous stem cell transplantation with pancytopenia: JACC patient care pathways. J Am Coll Cardiol.

[22] Laimoud M, Alanazi Z, Alahmadi F, Aldalaan A (2023). A challenging case of genetically and histologically diagnosed pulmonary veno-occlusive disease with extracorporeal life support and redo lung transplantation. Case Rep Cardiol.

[23] Kim ST, Xia Y, Tran Z, Hadaya J, Dobaria V, Choi CW, Benharash P (2022). Outcomes of extracorporeal membrane oxygenation following the 2018 adult heart allocation policy. PLoS One.

[24] De Carlis R, Buscemi V, Checchini G (2021). Liver transplantation from brain-dead donors on mechanical circulatory support: a systematic review of the literature. Transpl Int.

[25] Kim DH, Park JM, Son J, Lee SK (2021). Multivariate analysis of risk factor for mortality and feasibility of extracorporeal membrane oxygenation in high-risk thoracic surgery. Ann Thorac Cardiovasc Surg.

[26] Rubin J, Robinson E, Rubin EB (2023). The human and humanity that differentiate withholding from withdrawing life-sustaining therapy: an ECMO bridge to nowhere. Am J Bioeth.

[27] Stącel T, Urlik M, Antończyk R (2020). Extracorporeal membrane oxygenation as a bridge to lung transplantation: first Polish experience. Transplant Proc.

[28] Sanivarapu RR, Osman U, Latha Kumar A (2023). A systematic review of mortality rates among adult acute respiratory distress syndrome patients undergoing extracorporeal membrane oxygenation therapy. Cureus.

[29] Choi MC, Min EK, Yim SH (2023). Successful recovery after Veno-arterio-venous extracorporeal membrane oxygenation immediately before liver transplantation in multi-organ failure including acute respiratory distress syndrome: a case report. Transplant Proc.

[30] Wang B, Ye X (2024). Long-term lung function recovery after ECMO versus non-ECMO management in acute respiratory failure: a systematic review and meta-analysis. BMC Pulm Med.

[31] Leng A, Shou B, Liu O (2024). Machine learning from veno-venous extracorporeal membrane oxygenation identifies factors associated with neurological outcomes. Lung.

[32] (2024). ECMO Market Anticipates 4-6% Growth from 2024 to 2029. https://meditechinsights.com/extracorporeal-membrane-oxygenation-market/.

[33] Demartino ES, Braus NA, Sulmasy DP (2019). Decisions to withdraw extracorporeal membrane oxygenation support: patient characteristics and ethical considerations. Mayo Clin Proc.

[34] Gratz J, Pausch A, Schaden E (2020). Low molecular weight heparin versus unfractioned heparin for anticoagulation during perioperative extracorporeal membrane oxygenation: a single center experience in 102 lung transplant patients. Artif Organs.

[35] Lv X, Han Y, Liu D, Chen X, Chen L, Huang H, Huang C (2024). Risk factors for nosocomial infection in patients undergoing extracorporeal membrane oxygenation support treatment: a systematic review and meta-analysis. PLoS One.

[36] Aleksova N, Buchan TA, Foroutan F (2023). Extracorporeal membrane oxygenation for graft dysfunction early after heart transplantation: a systematic review and meta-analysis. J Card Fail.

[37] Lamb KM, Cowan SW, Evans N (2013). Successful management of bleeding complications in patients supported with extracorporeal membrane oxygenation with primary respiratory failure. Perfusion.

[38] Vajter J, Holubova G, Novysedlak R, Svorcova M, Vachtenheim J Jr, Vymazal T, Lischke R (2024). Anaesthesiologic considerations for intraoperative ECMO anticoagulation during lung transplantation: a single-centre, retrospective, observational study. Transpl Int.

[39] Hess NR, Hickey GW, Murray HN, Fowler JA, Kaczorowski DJ (2022). Ambulatory simultaneous venoarterial extracorporeal membrane oxygenation and temporary percutaneous left ventricular assist device bridge to heart transplantation. JTCVS Tech.

[40] Iglesias-Álvarez D, Pathania V (2021). LVAD as a bridge to decision complicated with pump thrombosis and infection. Indian J Thorac Cardiovasc Surg.

[41] Nunes-Carvalho J, Silva E, Spath P, Araújo-Andrade L, Troisi N, Neves JR (2025). Efficacy, safety, and complications of manta vascular closure device in VA-ECMO decannulation: a systematic review and meta-analysis. J Vasc Access.

[42] Zangrillo A, Landoni G, Biondi-Zoccai G (2013). A meta-analysis of complications and mortality of extracorporeal membrane oxygenation. Crit Care Resusc.

[43] Giraud R, Wozniak H, Donner V, Looyens C, Assouline B, Bendjelid K (2022). A dedicated expert ECMO-team and strict patient selection improve survival of patients with severe SARS-CoV-2 ARDS supported by VV-ECMO. J Clin Med.

[44] Godown J, Bearl DW, Thurm C (2019). Extracorporeal membrane oxygenation use in the first 24 hours following pediatric heart transplantation: Incidence, risk factors, and outcomes. Pediatr Transplant.

[45] Durães-Campos I, Costa C, Ferreira AR (2024). ECMO for drug-refractory electrical storm without a reversible trigger: a retrospective multicentric observational study. ESC Heart Fail.

[46] Jasseron C, Lebreton G, Cantrelle C (2016). Impact of heart transplantation on survival in patients on venoarterial extracorporeal membrane oxygenation at listing in France. Transplantation.

[47] Ius F, Aburahma K, Boethig D (2020). Long-term outcomes after intraoperative extracorporeal membrane oxygenation during lung transplantation. J Heart Lung Transplant.

[48] Rosenberg AA, Haft JW, Bartlett R, Iwashyna TJ, Huang SK, Lynch WR, Napolitano LM (2013). Prolonged duration ECMO for ARDS: futility, native lung recovery, or transplantation?. ASAIO J.

[49] Schmidt M, Bailey M, Sheldrake J (2014). Predicting survival after extracorporeal membrane oxygenation for severe acute respiratory failure. The Respiratory Extracorporeal Membrane Oxygenation Survival Prediction (RESP) score. Am J Respir Crit Care Med.

[50] Kowalewski M, Zieliński K, Gozdek M (2021). Veno-arterial extracorporeal life support in heart transplant and ventricle assist device centres. Meta-analysis. ESC Heart Fail.

[51] Warren A, Chiu YD, Villar SS (2020). Outcomes of the NHS England national extracorporeal membrane oxygenation service for adults with respiratory failure: a multicentre observational cohort study. Br J Anaesth.

[52] Iqbal MS, Wujcik KA, Nair A (2022). Long-term outcomes of ECMO post-heart transplant. J Heart Lung Transplant.

[53] DeFilippis EM, Clerkin K, Truby LK (2021). ECMO as a bridge to left ventricular assist device or heart transplantation. JACC Heart Fail.

[54] Delgado AA, Aguilar L, Kennedy K (2024). Association of ECMO flow index and mortality in adults with cardiogenic shock receiving venoarterial extracorporeal membrane oxygenation: an ELSO registry analysis. J Heart Lung Transplant.

[55] Organ Procurement and Transplantation Network. (2018). Policy 6 (2018). Organ Procurement and Transplantation Network (OPTN) | HRS. https://www.hrsa.gov/optn?from=optn.transplant.hrsa.gov.

[56] (2025). Transplant Candidates About the Adult Heart Allocation System FAQs | HRSA. https://www.hrsa.gov/optn/patients/resources/heart/heart-allocation-faqs.

[57] Peled Y, Ducharme A, Kittleson M (2024). International Society for Heart and Lung Transplantation Guidelines for the evaluation and care of cardiac transplant candidates-2024. J Heart Lung Transplant.

[58] (2025). ISHLT response to OPTN update on continuous distribution of hearts | ISHLT. https://www.ishlt.org/education-and-publications/resource/ishlt-response-to-optn-update-on-continuous-distribution-of-hearts.

[59] Egan TM, Murray S, Bustami RT (2006). Development of the new lung allocation system in the United States. Am J Transplant.

[60] Parker WF, Dussault NE, Jablonski R, Garrity ER, Churpek MM (2022). Assessing the accuracy of the lung allocation score. J Heart Lung Transplant.

[61] Rando HJ, Fanning JP, Cho SM, Kim BS, Whitman G, Bush EL, Keller SP (2024). Extracorporeal membrane oxygenation as a bridge to lung transplantation: practice patterns and patient outcomes. J Heart Lung Transplant.

[62] Keshavamurthy S, Bazan V, Tribble TA, Baz MA, Zwischenberger JB (2021). Ambulatory extracorporeal membrane oxygenation (ECMO) as a bridge to lung transplantation. Indian J Thorac Cardiovasc Surg.

[63] (2025). Amend status extension requirements in adult heart allocation policy. https://hrsa.unos.org/media/pufif23i/20250617_heart_data-report-2-year-monitoring-report-of-amend-status-extension-requirements.pdf.

[64] Otake M, Morita H, Sato K, Saku K (2025). Impact of venoarterial extracorporeal membrane oxygenation on hemodynamics and cardiac mechanics: insights from pressure-volume loop analysis. Int J Heart Fail.

[65] Shudo Y, He H, Elde S, Woo YJ (2023). Revised heart allocation policy improved waitlist mortality and waiting time with maintained outcomes in en-bloc heart-lung transplant candidates and recipients. Transpl Int.

[66] Parker WF, Chung K, Anderson AS, Siegler M, Huang ES, Churpek MM (2020). Practice changes at U.S. transplant centers after the new adult heart allocation policy. J Am Coll Cardiol.

[67] Moonsamy P, Axtell AL, Ibrahim NE (2020). Survival after heart transplantation in patients bridged with mechanical circulatory support. J Am Coll Cardiol.

[68] Mehta AB, Taylor JK, Day G, Lane TC, Douglas IS (2023). Disparities in adult patient selection for extracorporeal membrane oxygenation in the United States: a population-level study. Ann Am Thorac Soc.

[69] Ashana DC, Bhavsar NA, Viglianti EM (2023). Sociodemographic disparities in extracorporeal membrane oxygenation use: shedding light on codified systemic biases. Ann Am Thorac Soc.

[70] Borkowski P, Borkowska N, Mangeshkar S, Adal BH, Singh N (2024). Racial and socioeconomic determinants of cardiovascular health: a comprehensive review. Cureus.

[71] Butt SP, Razzaq N, Saleem Y, Cook B, Abdulaziz S (2024). Improving ECMO therapy: monitoring oxygenator functionality and identifying key indicators, factors, and considerations for changeout. J Extra Corpor Technol.

[72] Butt SP, Kakar V, Abdulaziz S (2024). Enhancing lung transplantation with ECMO: a comprehensive review of mechanisms, outcomes, and future considerations. J Extra Corpor Technol.

